# Asymmetric Membrane Capsules for Extended Delivery of the Weakly Basic Drug Carvedilol

**DOI:** 10.3390/pharmaceutics2020199

**Published:** 2010-05-18

**Authors:** Kumar Guarve, Ghanshyam Das Gupta

**Affiliations:** 1 Guru Gobind Singh College of Pharmacy, Yamuna Nagar, Haryana, India; 2 A.S.B.A.S.J.S.M College of Pharmacy, Bela, Punjab, India

**Keywords:** Extended release, asymmetric capsule, atenolol, pH regulating, fumaric acid

## Abstract

The objective of this study was to demonstrate that asymmetric membrane capsules can be used to deliver a poorly water soluble drug with a pH dependent solubility, such as carvedilol, for extended periods of time by modulating solubility with acid. In this study, the effect of the concentration of pH regulating agent and osmotic agents on the release rate of the active material was investigated. For this purpose, asymmetric membrane capsules of carvedilol were prepared using cellulose acetate as a semi-permeable membrane, containing glycerol as plasticizer, and fructose and fumaric acid were used as osmotic agent and pH regulating agent, respectively. In osmotic systems, the release rate of an excipient relative to the release rate of the drug is an important factor that determines the duration of drug release. Owing to high acidic strength and low aqueous solubility, fumaric acid resulted in simultaneous release and maintained a constant micro-environmental condition for the dissolution of the weakly basic drug. Finally, it was observed that the release rate of carvedilol was influenced by the concentration of fumaric acid and fructose. The optimal formulation was found to be able to deliver carvedilol at the rate of approximate zero-order up to 20 h, independent of release media and agitation rate.

## 1. Introduction

Utilization of osmotic pressure as a driving force for delivery of pharmaceutical agents in a controlled pattern for extended periods of time is a well-established fact [1]. Many different systems have been developed based on principles of osmotic pressure such as the elementary osmotic pump [2], the sandwiched osmotic tablet system [3], and push–pull osmotic systems [4]. For drugs that have low-solubility in the GIT, osmotically controlled delivery is more difficult, with elementary osmotic systems generally being considered unsuitable. While the push–pull osmotic and sandwiched osmotic tablet systems operate successfully in delivering water-insoluble drugs, they have a disadvantage compared with conventional immediate release tablets; the preparation of these systems is complicated. Not only do these system require a more complex bilayer press to prepare tablet cores, but also, stringent demands are placed on the properties of the two formulations being compressed together to form a cohesive core [5]. Use of asymmetric membrane coating in drug delivery has been described [6]. One advantage of osmotic capsules with asymmetrical membranes is that the membrane consists of a very thin, dense skin structure supported by a thicker, porous structural layer, and *in situ* formation of delivery orifices on this thin layer is potentially possible with no assistance [7]. Another advantageous characteristic of asymmetrical membranes in osmotic capsules is that these systems offer zero order release independence from pH, solubility and the speed of agitation, on the release rates and have shown good *in vivo–in vitro* correlation [8,9,10,11]. One of the advantages of an asymmetric membrane is the higher rate of water influx, allowing the release of drugs with a lower osmotic pressure or lower solubility. Carvedilol is a basic compound, used for the treatment of mild to moderate hypertension, angina pectoris, cardiac arrhythmias and myocardial infarction [12]. But the pH dependent solubility of carvedilol limits not only its bioavailability to 25–35%, but also formulation into desired dosage forms. Carvedilol’s extremely low solubility in alkaline pH may prevent the availability of the drug for absorption in the small intestine and colon, thus making it a poor candidate for an extended release dosage form. Thus, carvedilol, with significant solubility over the physiological pH, is an excellent candidate for investigating formulation strategies to overcome the pH-dependency. The incorporation of pH modifiers such as citric, fumaric or sorbic acid is a common approach employed with matrix and coated systems [13,14,15,16]. pH modifiers have been added to the core formulation in order to lower the micro-environmental pH inside the capsule and hence to keep the solubility of the drug substance independent from the pH of the aqueous medium. But in osmotic systems, the release rate of an excipient relative to the release rate of the drug is an important factor that determines the duration of drug release. Thus, highly water-soluble excipients are released much faster than the drug, which can limit their usefulness. Consequently, maintaining the desired pH over the entire period of drug dissolution is a challenging task. Keeping the above facts in view, it was thought of interest to use fumaric acid because of its high acidic strength and low aqueous solubility as a pH-modifier in the formulation to decrease the micro environmental pH of the core to a suitable level over the lifetime of the system, and thus to lead to a substantial increase in solubility of carvedilol. Therefore, fumaric acid was used as the solubility promoter to prepare carvedilol asymmetric membrane capsules in this study. The influences of fumaric acid and fructose on the drug release profile were investigated. The influences of release media and agitation rate on the *in vitro* release profile were also evaluated.

## 2. Experimental Section

### 2.1. Materials and methods

Cellulose acetate (Eastman Chem., USA), Glycerol, Acetone (E-Merck India Limited, Mumbai) were employed in preparation of shell membrane. Carvedilol was provided by N.B. Pharmaceutical Private Limited. Other chemicals used in preparation of buffers and other purpose were of analytical grade.

### 2.2. Solubility study

The solubility of carvedilol was determined in series of buffer solutions from pH range 1.2 to 7.0. Excess of drug was introduced into 25-mL stoppered conical flasks containing 10 mL of the respective solvent. The sealed flasks were agitated on a rotary shaker for 24 h at room temperature and equilibrated for 2 days. The aliquot were withdrawn and filtered through Whatmann filter paper No. 41, and the filtrate was suitably diluted and analyzed on an UV spectrophotometer at 284 nm.

### 2.3. Preparation of asymmetric membrane capsules

The asymmetric membrane capsules containing glycerol as pore forming agents were prepared by a dip coating method involving phase inversion. This process involves the precipitation of membrane structure on the stainless steel mould-pin by dipping the mould pin in a coating solution followed by quenching in aqueous solution to effect phase inversion. For this purpose, stainless steel mould-pins in shape of the capsule body (length 1.65 cm ± 0.03 cm and diameter 0.69 cm ± 0.024 cm) and cap (length 1.33 cm ± 0.034 and diameter 0.73 mm ± 0.037), were fabricated. The fabricated stainless steel mould pin were dipped into polymer solutions consisting of polymeric solution dissolved in the mixture composed of acetone, water, glycerin in various ratios (Table 1), for 2 min and then removed carefully so as to form a thin layer of coating solution on the mould pin. The pins were taken out of the coating solution and air dried for 60 seconds to allow evaporation of acetone present on the surface of the coat, followed by quenching in aqueous solution of 10% w/v glycerol for 5 min. This exchange of solvent with non-solvent resulted in phase inversion and formation of asymmetric membrane. After quenching time of 5 min the pins were removed and air-dried and the resulting asymmetric membrane in the shape of capsule body and cap were removed and air-dried for at least 24 h. After drying, they were trimmed and stored in desiccators for future study. 

**Table 1:** Composition of coating solution.

Ingredients	Coating Solution		
	F1	F2	F3
Cellulose acetate (w/v)	15%	15%	15%
Solvent system (Acetone/water)	45/5	45/5	45/5
Glycerol (w/w of CA)	50%	60%	70%

CA = Cellulose acetate.

### 2.4. Physical evaluation of asymmetric membrane capsules

#### 2.4.1. Weight variations

Twenty capsules were weighed individually. The average weight was calculated and was compared with the weight of each capsule.

#### 2.4.2. Surface characterization

Scanning electron micrograph of each capsule formulation was taken.

#### 2.4.3. Conformation of *in situ* pore formatio

The *in-situ* pore formation in asymmetric membrane capsules should take place due to the virtue of leaching of the pore forming agent present in the asymmetric membrane into the release medium. To confirm this phenomenon in the prepared system dye-test was conducted. The asymmetric membrane capsule with different concentrations of pore forming agent were filled with a highly water soluble amaranth dye (20 mg). The dye was filled in each of the capsule body manually and the cap was snugly fitted to the capsule body and finally sealed with a sealing solution of cellulose acetate only (14% w/v), to ensure that no release takes place from the seal. The capsules filled with dye were placed in 50 mL distilled water and observed for release of dye through the membrane. To demonstrate that the prepared system follows the osmotic principle to release its encapsulated contents, the capsules filled with amaranth dye were placed in a release medium of higher osmotic pressure (50 mL 10% w/v sodium chloride solution) and the capsules were visually observed for the release of dye.

### 2.5. Filling and sealing of asymmetric membrane capsules

The fabricated asymmetric membrane capsules of different porosity were filled with various ratios of drug: pH modifier and osmotic agent; to study the influence of pH modifier and osmotic agent on the release of poorly water soluble drug from the capsule shell. After filling, the cap was snuggly fitted on to the filled capsule body and finally the cap and body were hermetically sealed with a sealing solution of 14% w/v cellulose acetate only - without any pore forming agent - to ensure that no release takes place through the seal during the release rate study.

**Table 2:** Formulation of carvedilol asymmetric membrane capsules.

Formulation Code	Carvedilol	Fructose	Fumaric acid
F4	25	25	-
F5	25	125	-
F6	25	250	-
F7	25	250	25
F8	25	250	50
F9	25	250	100

### 2.6. In vitro *release rate study*

The asymmetric membrane capsules were subjected to an *in vitro* release rate study using USP dissolution test apparatus II (50 rpm, 37 °C ± 5 °C). 900 mL phosphate buffer (pH 7.4) was used as dissolution medium. Five milliliter of the sample was withdrawn hourly for 20 h, filtered through Whatmann filter paper No. 41 and suitably diluted with fresh dissolution medium and analyzed spectrophotometrically at λ_max_ = 284 nm. An equal volume of fresh dissolution medium, maintained at the same temperature, was added after withdrawing each sample to maintain the volume. The percentage of drug released at different time intervals was calculated using the equation generated from the standard curve.

## 3. Results and Discussion

Weight variations between the asymmetric membrane capsules and their dimensions were demonstrated to be consistent, with little variation. This confirms that the process of producing these capsules is reproducible. Membrane thickness and surface area (Table 3) were found to be almost the same for all the asymmetric membrane capsules, with slight variation. However, the weight of the capsules increased as the concentration of the pore forming agent was increased. The tensile strength of the asymmetric membrane of each of the batch increased with the increase in the concentration of pore forming agent as shown in Table 2. The void volume of each of the films was determined as a function of the volume of the pore-forming agent leached out of the membrane during the overnight dip in the vial resulting in void formation. A linear relationship between the concentrations of pore forming agent and void volume per weight of polymer were observed. This study confirms that void volume, thus the porosity of the membrane, would increase as the concentration of pore forming agent per unit weight of the polymer is increased.

**Table 3:** Average physical characteristics of the asymmetric membrane capsules.

Capsule shell code	F1	F2	F3
Membrane thickness (cm)	0.0273 ± 0.03	0.0244 ± 0.02	0.0224 ± 0.04
Surface area (cm^2^)	6.39 ± 1.5	6.41 ± 1.57	6.43 ± 1.4
Capsule shell weight (mg)	80.28 ± 1.96	85.37 ± 1.64	88.31 ± 1.73
Tensile strength (Kg/cm^2^)	0.119 ± 0.24	0.127 ± 0.83	0.188 ± 0.32
Void volume (cm^3^/g)	2.614 ± 0.32	3.949 ± 0.71	4.955 ± 0.17

**Figure 1:**
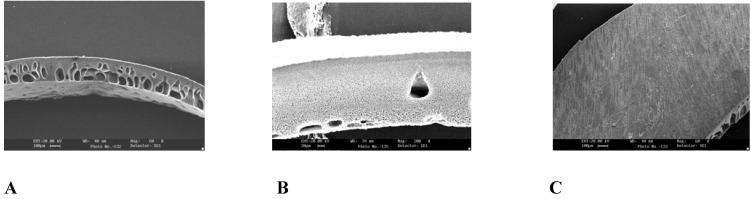
Scanning electron micrograph of the asymmetric membrane wall (A) cross section, at 60× magnification (B) porous region showing pores at 200× magnification (c) dense region showing no pores

Scanning electron micrographs of the capsule wall showed that the membrane was asymmetric with a relatively thin dense region on a porous substrate with longer microspores (Figure 1 A). No pore structures were shown in the dense region (Figure 1 C). The porous region at 200× magnification (Figure 1 B) reveals numerous pore structures. *In situ* formation of pores was proven, when a capsule encapsulated with amaranth was suspended in water medium, a deeply colored stream of amaranth was observed. This delivery process continued for another 30 min. However, when this capsule was suspended in a 10% NaCl solution, the osmotic effect was inactivated, and no release of amaranth was observed. This indicates that *in situ* formation of delivery orifice is possible in the thin structure of the asymmetric membrane, and also shows that the prepared system follows the principle of osmosis.

### 3.1. Solubility studies of carvedilol

Carvedilol exhibits pH dependent solubility. The measured solubility of carvedilol at 25 °C was 30 µg/mL, 70 µg/mL, 100 µg/mL and 18 µg/mL at pH 1.2, 4.5, 5 and 7, respectively. Thus, the solubility of carvedilol increases with decreasing pH and reaches a plateau near pH 5 and then starts decreasing.

### 3.2. *In vitro* release rate study

**Figure 2:**
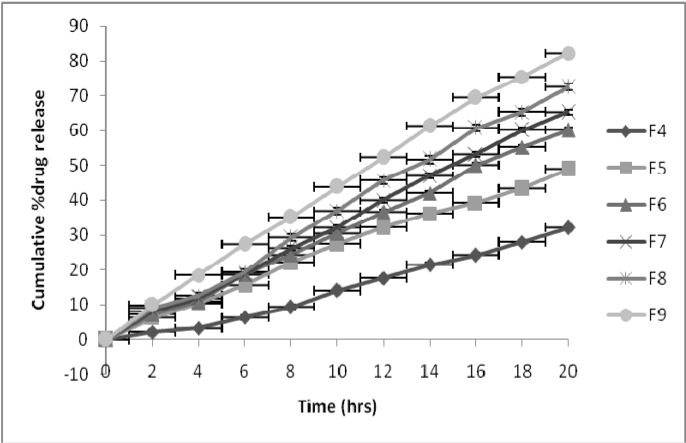
Dissolution of carvedilol from asymmetric membrane capsules.

The *in vitro* release of carvedilol from the asymmetric membrane capsule F1, F2 and F3 were studied. The release of carvedilol from these capsules was studied in the absence of pH modifier and osmotic agent. The drug was released from these systems by virtue of its own osmotic pressure. It was found that the amount of drug released increased as the concentration of pore forming agent was increased in the asymmetric membrane from 50% to 70%. The drug released from the capsule after 12 h with maximum pore forming agent F3 was only 10.46%, while it was 3.12% and 5.09% from the capsule F1 and F2, respectively. This increase in the amount of the drug released with the increase in concentration of pore forming agent suggests that the porosity of the asymmetric membrane increases significantly with the increase in the concentration of glycerol, causing a greater influx of dissolution medium in which the drug has appreciable solubility. Thus, the membrane with higher porosity would lead to early solubilization of the drug, causing its higher release. In spite of the increase in the porosity of the membrane, the amount of drug released by the virtue of its own osmotic pressure was found to be low. Hence, the rationale to study the effect of pH modifier and osmotic agents to increase the solubility and osmotic pressure of the core and the amount of drug released from the system were chosen. The release rate apparently increased with the increased amount of added fumaric acid and fructose. To optimize the amount of fumaric acid and fructose to be used in the formulation and to study the effect of drug-to-osmogent and pH modifier ratio, core formulations were prepared as shown in Table 3. It is clearly evident from Figure 2 that the higher release rates were observed from the systems containing pH modifying agent (fumaric acid), compared to systems filled with osmogent (fructose) alone. The increase in release rate may be due to the reduction of pH inside the capsule core; this enhances the dissolved amount of carvedilol in the core medium, thus increasing the osmotic pressure of the drug itself, resulting in an increased amount of drug being released from the system.

### 3.3. Evaluation of Optimal asymmetric membrane capsule formulation

**Figure 3:**
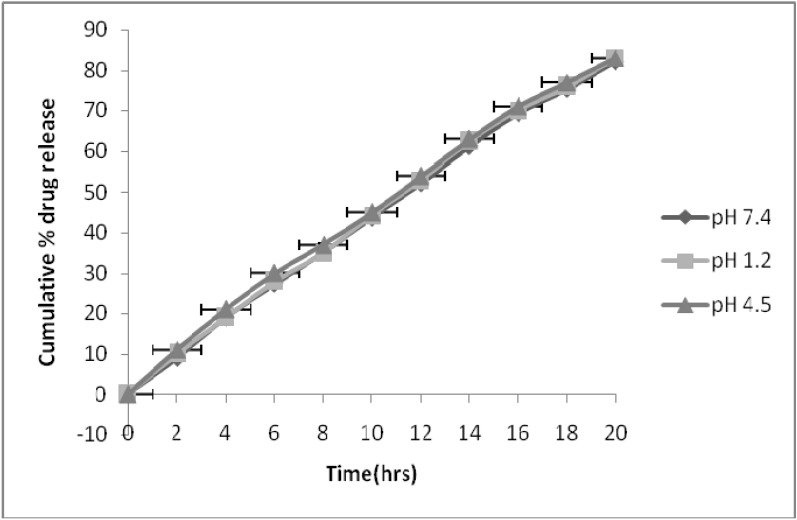
Effect of pH on drug release from optimized formulation.

Based on the results obtained, the optimal formulation was selected as follows: the weight ratio of carvedilol to fructose (1:10) and fumaric acid (1:4); percent of Glycerol in CA membrane was 70% (w/w of CA). To investigate the influence of release media on drug release, release tests of the optimal formulation were conducted in hydrochloric acid solution pH 1.2, phosphate buffer pH 4.5 and phosphate buffer pH 7.4. Figure 3 shows the release profiles in these release media. 

To further investigate the micro-environmental pH, the pH indicator methyl red (0.15% w/w) was added to the core to visually monitor the pH within the capsule during drug release. This indicator is red at acidic pH and yellow at pH values 5–8. After specific time intervals, the capsule was removed from the dissolution medium to visually monitor the pH within the core. The capsule core remained red (low pH). Thus, the pH within the core of the capsule remained acidic during drug release.

**Figure 4:**
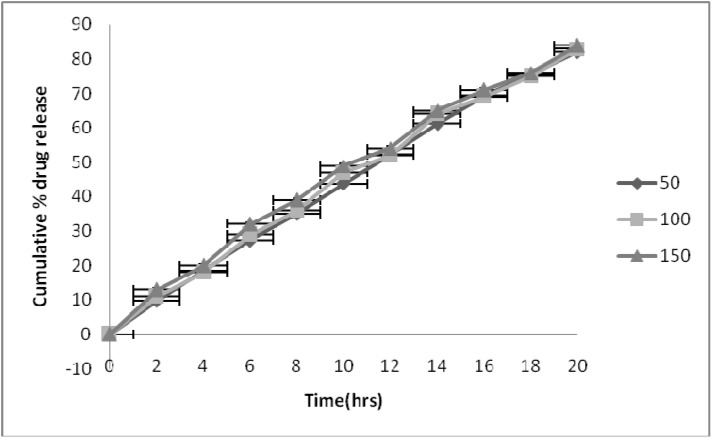
Effect of stirring rate on drug release from optimized formulation.

To study the effect of the stirring rate on the drug release profiles, dissolution tests of the optimal formulations were carried out at stirring rates of 50, 100 and 150 rpm. Figure 4 shows that an increase in the rate of stirring did not significantly affect the release rate of the drug. One way analysis of variance was used to assess the difference in release rate. Comparing the data of drug release in different media and at different agitation speeds, the p values obtained were 0.94 and 0.95, respectively, which was larger than 0.05, indicating that no significant differences existed in drug release in different release media and at different agitation speed. 

### 3.4. Kinetics of drug release

In order to understand the mechanism of drug release from the optimized system, the data were treated according to first-order (log cumulative percentage of drug remaining *versus* time) along with zero-order (cumulative amount of drug released *versus* time). When the data were plotted according to the first-order equation, the formulations showed a comparatively poor linearity, with regression value of 0.984, whereas the regression value for zero-order equation was 0.990, which indicated that drug release from the optimized formulation was independent of drug concentration.

**Table 4:** Kinetics of *in vitro* release of carvedilol from the asymmetric membrane Capsules.

Formulation code	Zero-order	First order
	R^2^	R^2^
F9	0.997	0.984
F8	0.996	0.972
F7	0.988	0.978
F6	0.998	0.983
F5	0.996	0.993
F4	0.988	0.979

## 4. Conclusions

The asymmetric membrane capsule of carvedilol using fructose as an osmotic agent and fumaric acid as pH modifier was studied. The optimal asymmetric membrane capsule was found to be able to deliver carvedilol at a rate of approximately zero order up to 20 h in pH 7.4, cumulative release at 20 h is 80%, independent of environment media and stirring rate. It is clear from the dissolution data that in order to deliver carvedilol over useful delivery duration, such as 20 h, the formulations must include a suitable osmotic agent, *i.e.,* one that has a high aqueous solubility and one pH modifying agent. Therefore, asymmetric membrane capsules can be used in the field of oral controlled release delivery of carvedilol.
